# Appropriateness of Web-Based Resources for Home Blood Pressure Measurement and Their Alignment With Guideline Recommendations, Readability, and End User Involvement: Environmental Scan of Web-Based Resources

**DOI:** 10.2196/55248

**Published:** 2025-04-03

**Authors:** Eleanor Clapham, Dean Picone, Samuel Carmichael, Carissa Bonner, Niamh Chapman

**Affiliations:** 1 School of Health Sciences Faculty of Medicine and Health University of Sydney Sydney Australia; 2 Menzies Institute for Medical Research University of Tasmania Hobart Australia; 3 Menzies Centre for Health Policy & Economics School of Public Health University of Sydney Sydney Australia

**Keywords:** readability, online resources, blood pressure guidelines, end user, home blood pressure measurement, patient education, educational resource, self-education, hypertension

## Abstract

**Background:**

High blood pressure (≥140/90 mm Hg) is the most prominent mortality risk factor worldwide. Home blood pressure measurement (HBPM) is recommended for blood pressure (BP) management. HBPM is most effective to improve BP management when delivered with patient education. It is unknown whether web-based resources are appropriate for patient education for HBPM. Patient education should provide accurate, evidence-based information, communicate at an eighth grade reading level, and involve end users in development to meet the needs of adults of all health literacy levels. Using these criteria, this study aimed to determine the appropriateness of web-based HBPM resources.

**Objective:**

This study aimed to determine whether web-based resources are appropriate for HBPM education based on three research questions: (1) Do web-based resources provide evidence-based information that aligns with guideline recommendations? (2) Do they communicate at an appropriate reading level? (3) Do they involve end users in their development?

**Methods:**

An environmental scan of web-based resources for HBPM was conducted on Google (October 2022) using search terms developed with consumers (n=6). Resources were included if they were identified on the first page of the search findings, not paywalled, and in English. Resource appropriateness was appraised based on three criteria: (1) alignment of resource content to 23 recommendations for HBPM from 6 international guidelines, (2) being at an appropriate grade reading level as determined by a health literacy assessment software, and (3) having evidence of end user involvement in resource development.

**Results:**

None of the identified resources (n=24) aligned with all 23 of the guideline recommendations. All resources aligned with the recommendation to measure BP when seated, while few aligned with the recommendation to use a validated BP device (n=9, 38%). All resources exceeded the recommended eighth grade reading level (mean 11.8, range 8.8-17.0) and none reported evidence of patient end user involvement in development.

**Conclusions:**

None of the web-based resources met the criteria for appropriate education to support adults to measure BP at home. Resources should be developed with end users using health literacy tools and multimodal communication methods to ensure they are appropriate to meet the needs of patients.

## Introduction

High blood pressure (BP; hypertension: BP≥140/90 mm Hg) is the leading risk factor for death worldwide [1,2]. High BP can be controlled (<140/90 mm Hg) via medication and lifestyle changes to reduce the risk of heart attack and stroke [3]. Home BP measurement (HBPM) is widely recommended to inform hypertension diagnosis and to monitor the control and ongoing management of BP [4-7]. HBPM provides accurate, standardized BP readings, which have greater prognostic value for cardiovascular disease when performed according to guideline recommendations [8]. Adults who measure BP at home are more engaged in BP management and achieve greater BP control [9]. Further, several studies, including a recent meta-analysis, highlight that HBPM is only effective for improving BP management when accompanied by appropriate patient education on how to measure HBPM accurately and act on BP readings [7,10,11]. However, there is a lack of guidance and standardized resources to provide effective education for HBPM in clinical settings [11,12].

In the absence of effective in-clinic education and with the increased use of telehealth, web-based resources are commonly used by adults who seek health-related information for self-education [13]. In addition, recent work in Australia has shown that >35% of adults would prefer to access information about high BP on the web [14]. With this evolution of health care and patient education delivery, government bodies have emphasized the need for web-based resources to provide health information that is evidence-based and understandable [15]. More specifically, several systematic reviews on eHealth, mobile health, and other digital strategies to improve BP management also suggest a growing need to ensure that appropriate education is available on the web to support adults to undertake HBPM [16-18].

Web-based educational resources that are appropriate to support adults to perform HBPM should deliver evidence-based information in a manner that meets the health literacy and learning needs of most adults. To do this, information should be presented at an eighth grade reading level, with the use of visual aids such as graphics to support understandability [19,20]. The use of co-design methods involving target end users during resource development is an effective method to ensure that resources meet the needs of end users for effective patient education [21-24]. However, previous research has shown that web-based educational resources for cardiovascular disease risk management do not provide appropriate information or meet the usability or readability needs of adults [18,25-29], and co-design involving end users (such as community members and medical professionals) is an underused method during resource development [24]. Due to the importance of patient education for HBPM to achieve improved BP control and patient self-efficacy in BP management, patient education resources for HBPM should be appropriate for use by adults who self-monitor BP.

The aim of this study was to determine whether web-based resources are appropriate for HBPM patient education based on three research questions: (1) Do web-based resources provide evidence-based information that aligns with guideline recommendations? (2) Do they communicate at an appropriate reading level? (3) Do they involve end users in their development?

## Methods

### Study Design

An environmental scan of web-based resources on HBPM was conducted through a Google search designed to emulate the approach taken by adults with lived experience of HBPM when seeking web-based material about HBPM [27]. The resources were characterized according to basic identifying features such as publishing organization and year. Resources were assessed for alignment with 23 recommendations common across 6 international guidelines that encompass HBPM activities including acquiring the BP measurement device, scheduling and preparing for HBPM, selecting and fitting the cuff, BP measurement conditions, and recording and reporting BP readings (Textbox 1 and Multimedia Appendix 1) [6,30-34]. The grade reading level of the content of the resources was determined using the health literacy assessment software Sydney Health Literacy Lab Editor (SHeLL Editor) [19,35,36]. The recommended reading level for maximum comprehension for adults is eighth grade or below [19]. Involvement of community member and/or medical professional end users in resource development was assessed according to whether this was reported within each resource. Data extraction and resource appraisal were undertaken by 2 independent researchers (EC and SC) using a coding framework hosted on the secure web-based platform REDCap (Research Electronic Data Capture; Vanderbilt University) [37].

**Twenty-three key guideline recommendations for home blood pressure measurement.**

**Acquiring the blood pressure (BP) measurement device:**

- Use a validated BP measurement device for home BP measurement (HBPM).
- Finger cuff BP measurement devices should not be used for HBPM.

**Scheduling HBPM:**

- On a day that HBPM is being conducted, BP should be measured in the morning and the evening.

**Preparing for HBPM:**

- Do not measure BP if uncomfortable, stressed, or in pain.
- Measure BP before medication.
- Measure BP before eating or 30 minutes or 2 hours after eating.
- Measure BP after emptying the bladder.
- Measure BP before exercise or 30 minutes after exercise.
- Measure BP before consuming caffeine or after 30 minutes or 1 hour of consuming caffeine.
- Measure BP before smoking or 30 minutes or 1 hour after smoking.
- Have 5 minutes, or at least 5 minutes, of seated rest before measuring BP.

**Selecting and fitting the cuff:**

- Use an appropriately sized arm cuff for HBPM.
- The arm cuff should fit the arm within the accepted range indicated on the cuff.
- Fit the upper arm BP cuff to a bare arm.

**Measurement conditions:**

- Measure BP in a room at a comfortable temperature.
- Measure BP with the arm fitted with the cuff supported or supported at the heart level.
- Measure BP in a seated position.
- Measure BP with both feet flat on the floor.
- Measure BP with legs uncrossed.
- Measure BP with back supported.
- Take 2 readings 1 minute apart at each HBPM sitting.

**Recording and reporting BP:**

- Average the BP readings taken over a 7-day period, discarding the first day.
- Take a copy of home BP readings to a doctor.

### Search Strategy

The search engine Google Australia was used to identify web-based resources addressing HBPM. Seven search terms were developed with trained research consumer advisors who have lived experience of BP management and using Google Trends (Multimedia Appendix 2). Consumer advisors (n=6) identified the search engine and the top 5 search terms they would use to find information about HBPM on the web. Google Trends was used to identify the search queries related to the term “home blood pressure measurement,” which had the highest probability of use worldwide on Google from January 1, 2012, to October 7, 2022. Search terms suggested by consumer advisors, which also had high probability of use on Google according to Google Trends and were relevant to HBPM were used. Search terms included the following: “How to take your blood pressure,” “How to check blood pressure at home,” “How to take blood pressure at home,” “Home blood pressure monitoring,” “How to measure blood pressure at home,” “How to monitor blood pressure at home,” and “Home blood pressure measurement.”

### Data Extraction From Web-Based Resources

Data extraction was undertaken independently by 2 investigators (EC and SC) on October 17, 2022 (duplicate search). To avoid potential bias attached to the reviewers’ Google history, each reviewer conducted the search using default Google search settings within the incognito browser of Google Chrome and cleared the cache before each search. The results obtained with each search term, which were present on the first page of the search findings on Google, were exported, excluding advertisements. After completing all searches, the resources extracted by each reviewer across all search terms were combined, and duplicate resources were removed (ie, resources identified across >1 search term).

### Inclusion Criteria for Web-Based Resources

HBPM resources were included if they met the following inclusion criteria: (1) they were free to access by the public (eg, no paywalls), (2) they were available in English, and (3) they contained content relevant to HBPM (eg, resource mentions “home blood pressure measurement” or “self-measured blood pressure”; Multimedia Appendix 3). The resources extracted from Google were independently analyzed against inclusion criteria by EC and SC, and discrepancies were resolved by third and fourth independent reviewers based on the same criteria (NC and DP). All resources that met the inclusion criteria were included for analysis. Resources were not excluded due to criteria regarding publication date, publication location, or resource format (ie, video, graphic, or blog).

### Appraisal of Web-Based Resources

A coding framework hosted on REDCap was used by EC and SC to independently and systematically appraise resources according to three criteria: (1) alignment of resource information with HBPM guideline recommendations, (2) grade reading level of the content of the resources, and (3) end user involvement in resource development. The REDCap appraisal framework captured resource characteristics (type of publishing organization, authorship, year of publication or last review, and location of publication and languages), communication methods used (categorized as written text, visual, video, or audio), the alignment of resource content against HBPM recommendations, and the grade reading level of the content of the resources (Multimedia Appendix 3). Independent reviewers were trained on how to undertake the search, extract data, and appraise resources. During training, the appraisal data of a subset of resources (n=3) were compared to ensure that the correct process was undertaken by both independent reviewers. All data were captured in a framework housed on REDCap. All content (including audio, text, video, and graphical content) included within each HBPM resource was appraised according to 3 main criteria detailed below. Any discrepancies in appraisal were resolved in adjudication sessions where blinded discrepancies were presented to adjudicators (NC and DP) and resolved via discussion until consensus was reached.

### Alignment of Resource Information With HBPM Guideline Recommendations

Twenty-three recommendations that encompass activities for HBPM from 6 international guidelines were used to determine the alignment of resource content to the guidelines (Textbox 1) [6,30-34]. Resource content was marked against each recommendation and categorized as “aligned with” if the resource correctly stated the recommendation, “incorrectly stated” if the resource incorrectly or incompletely stated the recommendation, or “not mentioned” if the resource did not include the recommendation (Multimedia Appendix 3). Where resources “incorrectly stated” a guideline recommendation, the incorrect information provided by the resource was recorded on REDCap (Multimedia Appendix 4).

### Grade Reading Level of Resource Text

The grade reading level of each resource was calculated using the SHeLL Editor, which is a health literacy assessment tool that calculates the school grade reading score of text according to the Simple Measure of Gobbledygook, and reports other measures such as complex language, uncommon English words, and the use of passive voice [19,35,36]. All text presented within each resource (including written text, image captions, and audio and video transcripts when available) was entered into the SHeLL Editor and the grade reading level, and associated measures were recorded in the REDCap framework (Multimedia Appendix 3).

### End User Involvement in Resource Development

End user involvement in resource development was recorded in the REDCap framework as stated within the resource. End users were defined as adults who seek information to measure BP at home (eg, patient, health consumer, service user, carer, or community advisor) or medical professionals due to their role in delivering education for HBPM to adults or directing adults to educational resources for HBPM [38,39].

### Data Analysis

Data were analyzed using Stata (version 17; StataCorp). Resources were assigned an identifying number for analysis and the presentation of results (Multimedia Appendix 5). Categorical data are presented as n (%) values.

## Results

### Resource Characteristics

Twenty-four resources were included in the study (Multimedia Appendices 5 and 6). Not-for-profit organizations (such as the National Heart Foundation) were the most common type of publishing organization (n=6, 25%) followed by websites (such as Healthline), academic journals, and scientific societies (Table 1). Most resources were communicated via a combination of written text, visual (eg, images), and audio and video communication methods (n=17, 71%), and the remaining resources were communicated by written text only (n=7, 29%; Table 1). Most resources were published in Australia (n=10, 42%) or North America (n=9, 38%), and only 3 (13%) were available in languages other than English.

**Table 1:** Characteristics of the included resources.

Characteristic			Resources, n (%)
**Type of publishing organization**			
	Commercial entity	3 (12)	
	Scientific journal	4 (17)	
	Government body	1 (4)	
	Not-for-profit organization	5 (21)	
	Scientific society	5 (21)	
	Website	4 (17)	
	Medical research institute	2 (8)	
**Date of publication or last review**			
	Last 12 months	3 (13)	
	1-2 years ago	3 (13)	
	2-3 years ago	2 (8)	
	3-4 years ago	1 (4)	
	4-5 years ago	1 (4)	
	>5 years ago	4 (16)	
	Not stated	10 (42)	
**Location of publication**			
	Australia	10 (42)	
	North America	9 (37)	
	Europe	5 (21)	
**Communication method**			
	Written text only	7 (29)	
	Written text and visual	9 (37)	
	Written text and video	4 (17)	
	Written text, visual, and video	3 (12)	
	Written text, audio, and visual	1 (4)	
**Languages**			
	English only	21 (88)	
	English, Mandarin, and Spanish	1 (4)	
	English and Spanish	2 (8)	

### Resource Alignment With HBPM Guideline Recommendations

**Figure 1:**
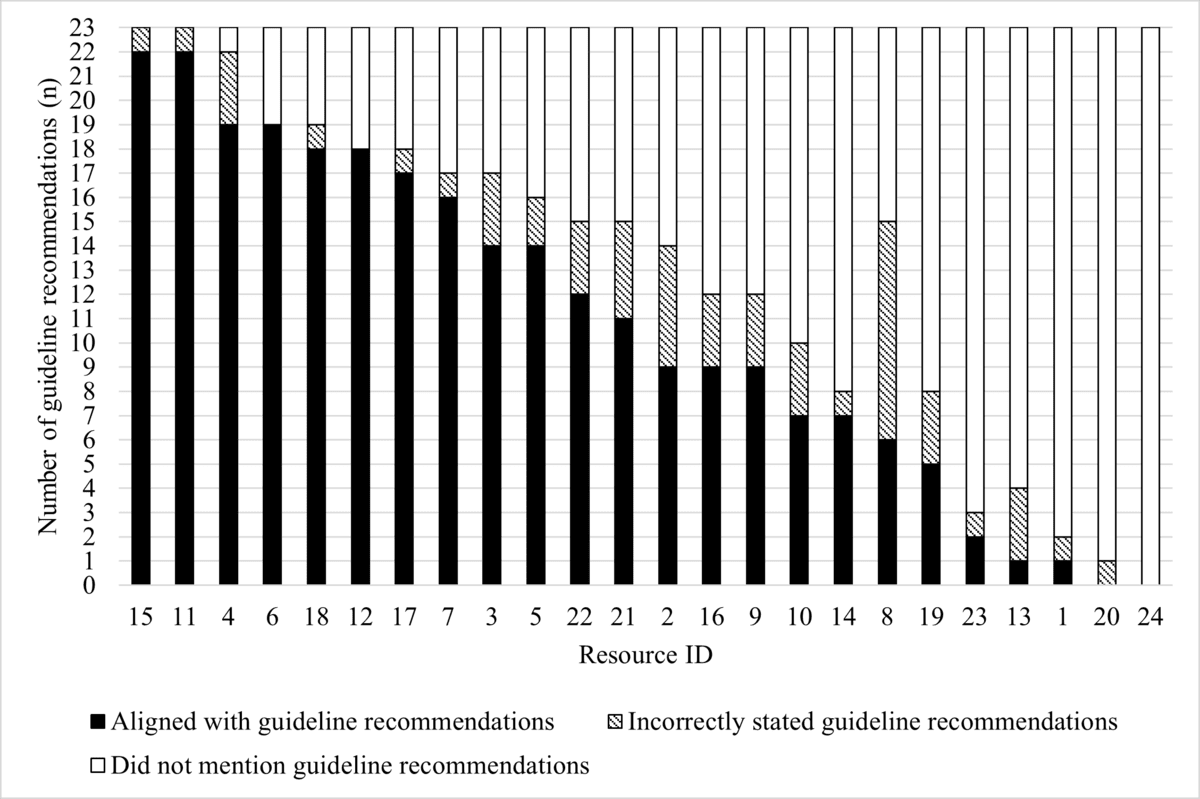
Alignment of each resource to 23 guideline recommendations for home blood pressure measurement. Resource content either "aligned with" the HBPM guideline recommendations (black bars), "incorrectly stated" the guideline recommendations (patterned bars), or "did not mention" the guideline recommendations (white bars). The y-axis indicates each of the 23 guideline recommendations, and the x-axis indicates the number of resources (n=24 resources).

**Figure 2:**
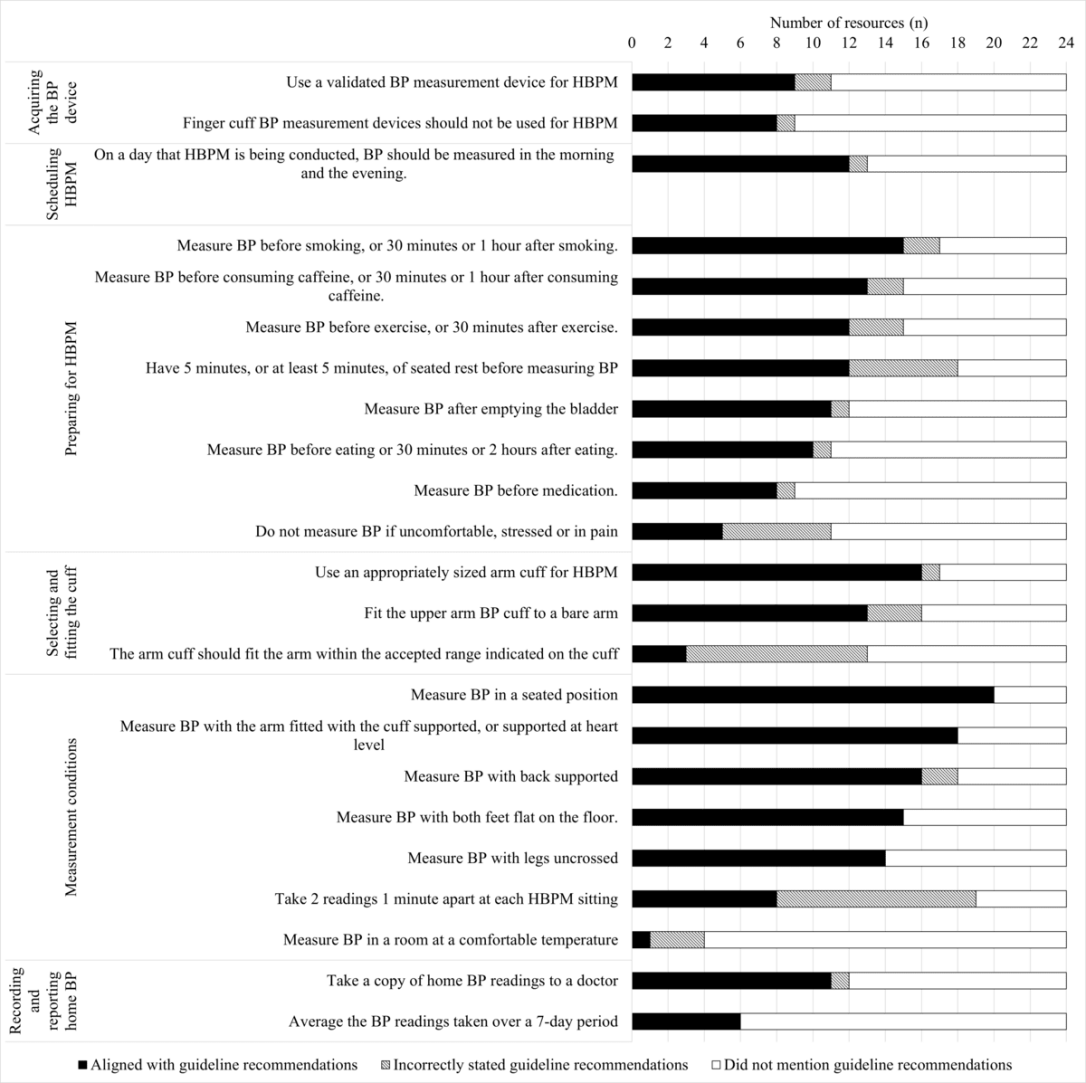
Alignment of all resources to each of the 23 guideline recommendations for key home blood pressure measurement (HBPM) activities. Resource content either "aligned with" the HBPM guideline recommendations (black bars), "incorrectly stated" the guideline recommendations (patterned bars), or "did not mention" the guideline recommendations (white bars). The x-axis indicates the number of HBPM resources. BP: blood pressure.

As shown in Figure 1, none of the resources aligned with all 23 guideline recommendations for HBPM. Almost all (n=22, 92%) of the resources incorrectly stated at least one guideline recommendation. Two (8%) resources did not align with any of the 23 guideline recommendations for HBPM. The alignment of resources with each guideline recommendation is shown in Figure 2, indicating whether the recommendation was “aligned with,” “incorrectly stated,” and “not mentioned” in each resource. Time- or frequency-bound recommendations were often incorrect within resources. For example, to rest for 5 minutes before measuring BP was incorrectly stated in 25% (n=6) of resources and to take 2 BP readings 1 minute apart at each sitting was incorrect in 46% (n=11) of resources (Figure 2).

Resources incorrectly stated guideline recommendations because their content was not specific enough to capture the meaning of the guideline recommendation, provided contradictory advice, or stated an alternate rest period, number of measurements, frequency, duration, or other numeric parameters to the guideline recommendations (Multimedia Appendix 4). For example, rather than stating the recommendation to “have five minutes [or at least five minutes] of seated rest before measuring BP,” resources that incorrectly stated this recommendation said to “rest for 15 minutes” (resource ID 10) or “rest quietly and wait about one to two minutes before taking another measurement” (resource ID 19). In addition, rather than stating the recommendation to “take two readings one minute apart at each HBPM sitting,” a resource that incorrectly stated this recommendation said “if you get a reading that is slightly or moderately higher than normal, take your blood pressure a few more times” (resource ID 6).

**Figure 3:**
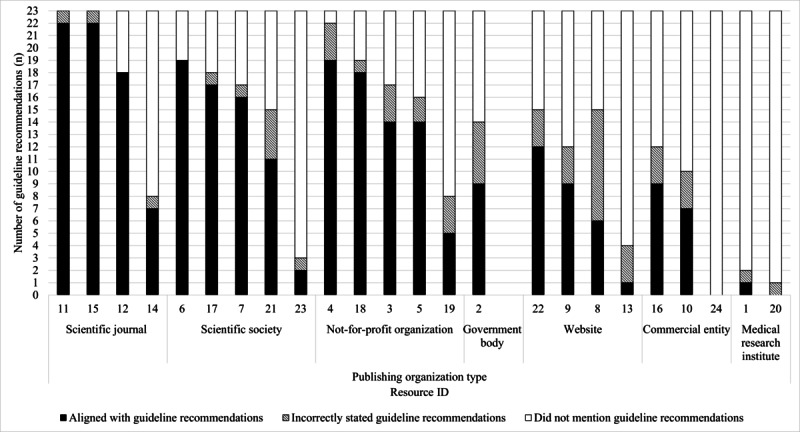
Resource alignment to home blood pressure measurement (HBPM) guideline recommendations according to the type of publishing organization. Resource alignment was determined by appraising resource content against 23 guideline recommendations of core HBPM activities. Resource content either "aligned with" the HBPM guideline recommendations (black bars), "incorrectly stated" the guideline recommendations (patterned bars), or "did not mention" the guideline recommendations (white bars).

Resource alignment to guideline recommendations according to the publishing organization is outlined in Figure 3. Resources published by scientific journals, scientific societies, and not-for-profit organizations aligned with a higher number of HBPM guideline recommendations (14 resources; median 16.5, range 2-22 recommendations) than resources published by websites, commercial entities, and medical research institutes (9 resources; median 6.5, range 0-12 recommendations; Figure 3).

### Grade Reading Level of Resource Text

**Figure 4:**
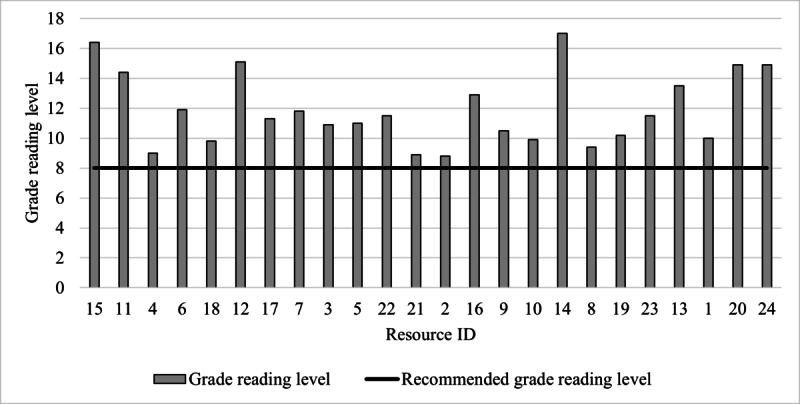
Grade reading level of web-based home blood pressure measurement (HBPM) resources. Resource grade reading level (y-axis) is presented in the order of resources from the highest (left) to the lowest alignment (right) with HBPM guideline recommendations. The grade reading level (y-axis) of resource content was calculated by inputting all resource content into the Sydney Health Literacy Lab Editor.

**Figure 5:**
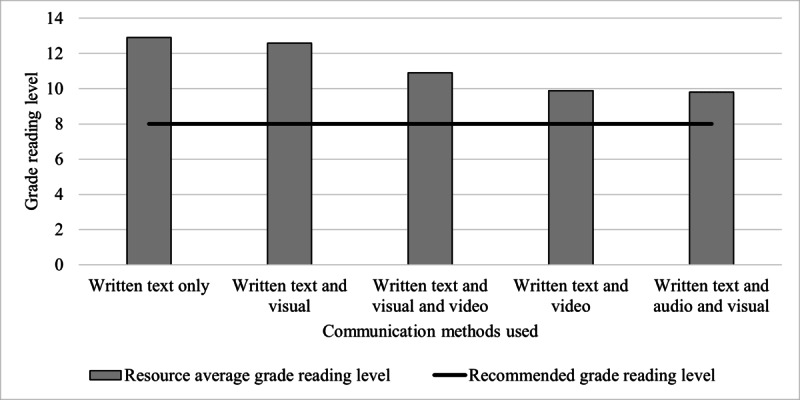
Resource grade reading level according to the communication method. The average grade reading level (y-axis) of resources according to communication methods used in the resource (x-axis). The grade reading level (y-axis) of resource content was calculated by inputting all resource content into the Sydney Health Literacy Lab Editor.

**Figure 6:**
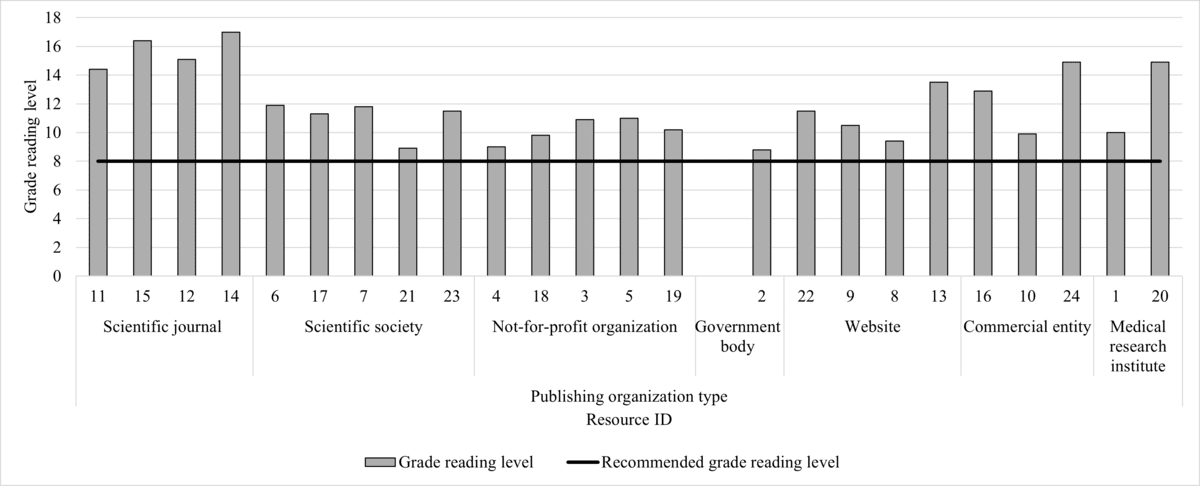
Resource grade reading level according to the type of publishing organization. Grade reading level was calculated by inputting all resource content into the Sydney Health Literacy Lab Editor.

All resources exceeded the recommended eighth grade reading level (grade reading level: mean 11.8, range: 8.8-17.0; Figure 4). The grade reading level of resources did not differ according to the level of alignment with HBPM guideline recommendations or communication methods used (Figures 4 and 5). Resources presented through written text only (n=7) had the highest average grade reading level (grade reading level: mean 12.9, range 10.5-16.4; Figure 5). Resources published by scientific journals had the highest average grade reading level (n=4; grade reading level: mean 16.5, range 11.9-17), compared to government bodies (n=1; grade reading level: mean 8.8) and not-for-profit organizations (n=5; grade reading level: mean 10.2, range 9-10.9; Figure 6), which had the lowest average grade reading levels. Multimedia Appendix 7 shows the characteristics of the resource text that contributed to the grade reading level score.

### End User Involvement in Resource Development

**Figure 7:**
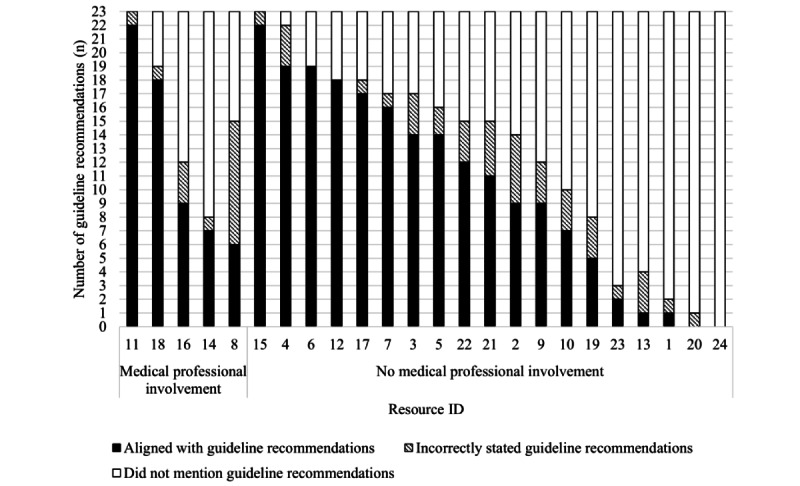
Resource alignment to guideline home blood pressure measurement (HBPM) recommendations according to medical professional involvement during resource development. Resource alignment was determined by appraising resource content against 23 guideline recommendations of core HBPM activities. Resource content either "aligned with" the HBPM guideline recommendations (black bars), "incorrectly stated" the guideline recommendations (patterned bars), or "did not mention" the guideline recommendations (white bars).

**Figure 8:**
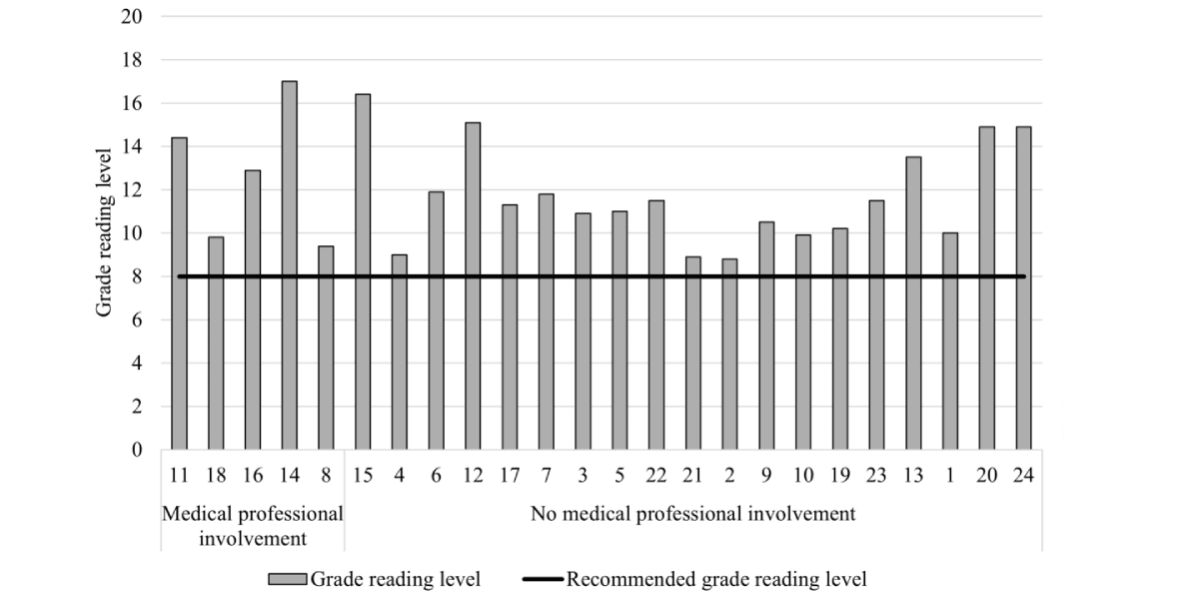
Resource grade reading level according to medical professional involvement during resource development. Grade reading level was calculated by inputting all resource content into the Sydney Health Literacy Lab Editor. Resources are presented in order of the highest (left) to the lowest alignment (right) to home blood pressure measurement guideline recommendations.

None of the resources reported involving adults (such as patients, health consumers, or carers) during resource development. Medical professional involvement was reported in 5 (21%) resources. Resources with and those without medical professional involvement during development had similar alignment with HBPM guideline recommendations and grade reading levels (Figures 7 and 8).

## Discussion

### Principal Findings

This study has demonstrated that web-based resources may not be appropriate to fully support adults to undertake high-quality HBPM because none of them provided sufficient guideline information or communicated at an appropriate reading level. Using methodology that emulated the search strategy of adults with lived experience of HBPM to identify web-based resources, we identified that none of the resources correctly stated all key guideline recommendations for HBPM, and most resources included information that was incorrect according to guideline recommendations due to incorrectly stating time- and frequency-bound recommendations. The findings of this study highlight the need to design educational materials for key BP management behaviors such as HBPM, which are appropriate for adults who self-monitor BP.

Calls to action on hypertension in the United States and Australia highlight the importance of empowering patients who perform HBPM to improve and monitor BP control [40,41]. At a global level, the World Heart Federation Hypertension Roadmap highlighted that appropriate patient education is an important strategy to improve BP control [20]. Existing research supports this by illustrating that patient education delivered with HBPM improves BP control outcomes [10] and adherence to recommendations for HBPM [11,42]. However, the results of this study suggest that web-based resources may not be appropriate to educate adults about HBPM as the identified resources did not provide guideline aligning information required to support HBPM in a manner that met adult reading needs.

This study found that time- and frequency-based HBPM recommendations, such as the number of BP measurements to take per sitting and the associated rest periods, were incorrectly stated within the most resources, while the recommendation to measure BP when seated was accurately communicated in the most resources. Interestingly, a recent study on BP guideline recommendations followed by adults who measure BP at home found that time-bound recommendations were adhered to by the lowest number of adults, while the recommendation to measure BP when seated was performed by the highest number of adults. Additionally, adults who reported to have previously sought information to support HBPM did so via web-based sources; however, these adults did not perform higher-quality HBPM than those who had not used web-based information to support HBPM [43]. Altogether, these findings highlight that current educational resources are not appropriate to support adults to measure BP at home as recommended by guidelines and illustrate a possible synergy between the inaccurate information delivered within web-based HBPM resources and the practice of adults when measuring BP at home. This emphasizes the need for web-based HBPM resources to accurately and clearly deliver guideline recommendations to enable proper HBPM practice among adults, which is an important behavior for BP management.

This study found that resources published in scientific journals, scientific societies, and not-for-profit organizations stated more guideline recommendations correctly than resources published by websites, commercial entities, and medical research institutes. This suggests that some organizations and resource developers may have low awareness of or access to guideline recommendations for HBPM and may not recognize the importance of standardized BP measurement practices for achieving and maintaining BP control. International BP guidelines should consider the importance of using consistent, unambiguous, and plain language for HBPM recommendations to support the accurate translation of recommendations into educational resources for HBPM. To ensure that guideline information is disseminated to the general public, guideline developers should share guidelines with organizations that publish health information on the web and partner with peak organizations to enable resource developers from outside of the scientific and clinical community to create guideline-informed, evidence-based resources.

Apart from correctly delivering evidence-based guideline information, HBPM resources must deliver information in a format accessible for adults to achieve effective education. Previous evidence has shown that web-based health information is not appropriate to inform patient decisions surrounding cardiovascular disease because the reading level is too high and the information is not adapted to meet the learning needs of adult patients [17,26,27,44]. This is consistent with the findings of our study where all resources were at a reading level that was too high (≥8 grade) for adult comprehension and over a quarter (n=7, 29%) of resources only presented information via written text only.

Strategies to deliver patient education that meet the literacy levels of adult patients should be implemented to ensure that educational resources can support adults to perform key cardiovascular disease risk management behaviors such as HBPM. As highlighted by the World Heart Federation Hypertension Roadmap, the delivery of education via graphical means is a more appropriate communication method to meet the needs of those with lower health literacy levels [20]. This is supported by the findings of this study, where web-based resources with multimodal communication methods achieved a lower average grade reading level than those that communicated via written text alone. Supporting audiovisuals, such as graphs, diagrams, images, videos, and the read-aloud function should be used to aid understandability, comprehensibility, and actionability of web-based health information. Additionally, the use of readability and grammar editing tools when developing resources may help to ensure that resource information is presented at a grade reading level that is appropriate to all adults, and resources such as the Agency for Healthcare Research and Quality’s Health Literacy Universal Precautions Toolkit may provide actionable methods to maximize the understandability of patient education strategies [45]. Finally, artificial intelligence (AI) could be used to tailor web-based information to meet patient literacy needs, selectively deliver information most relevant to the unique information needs of patients, and support chat box functions enabling adults to ask clarifying questions [46]. However, although AI-generated content is accurate and retains key meaning, caution should be exercised to ensure that information used by AI generators is sourced from guidelines.

Direct end user involvement in resource development is an increasingly well-recognized strategy to ensure that health products and services, including health information, meet end user needs to deliver quality care and education [21-24]. However, end user involvement is not commonplace in resource development [24], which is consistent with the findings of our study. Although some resources of this study involved medical professionals in their development, this did not improve the resource grade reading level or the number of correctly stated guideline recommendations. While medical professionals play a central role in patient education, they may not be aware or have sufficient resources to meet the health literacy needs of all patients [47-49]. Additionally, some medical professionals have general distrust in BP guidelines [50] and do not use current guidelines recommendations for HBPM in clinical practice, such as the recommendation to use different cutoffs for a hypertension diagnosis using in-clinic versus at-home BP readings [12,51]. This further highlights the need for adults with lived experience of BP management to be involved in resource development to identify unfamiliar medical jargon, recommend culturally and linguistically sensitive adaptations, and advise on the appropriate use of images. For existing resources, such as those identified in this study, adults could be involved in appraising these resources to identify how they could better meet the needs of adults seeking information on HBPM. Implementing the strategies suggested would ensure that information provided by web-based resources is suitable for use by all end users to support high-quality HBPM among adults.

### Strengths and Limitations

A strength of this study was the involvement of consumer advisors in the development of the search strategy to emulate the experience of adults seeking information for HBPM. In addition, a rigorous framework analysis approach was used by 2 independent researchers for resource identification and appraisal. This study was strengthened by the guideline-informed appraisal process; however, guideline recommendations included in this analysis were not exhaustive of all recommendations for HBPM due to inconsistency in recommendations across guidelines. The incognito mode was used to eliminate the impact of cookies and search history unique to the reviewer. However, as a result of using default Google search engine settings and including only the first page of search results, some web-based HBPM resources would have been missed. The location at which this study was conducted has likely impacted the search results, as 42% of included resources were from Australia. This suggests that the resources that an adult seeking HBPM information is exposed to depends on the location from which the search is conducted. This method should be replicated in other locations to assess resources that may not have been identified in this study. The scope of this study was narrow, with highly specific appraisal criteria used to evaluate resources. Other important considerations of web-based resources such as ease of access and usability should be included in future studies for a more complete understanding of resource appropriateness to support HBPM. Further, given the proliferation in use of AI, mobile health, and eHealth for health interventions and patient education, HBPM resources found on these information sources should also be appraised for appropriateness.

### Conclusion

This study found that the web-based resources identified herein are not appropriate to fully support adults to measure their BP at home according to HBPM guideline recommendations. None of the resources identified provided sufficient guideline information to support adults to perform high-quality HBPM, were presented at an appropriate reading level, or involved end users in their design. Resources that deliver health information should use strategies such as the use of multimodal communication methods, literacy editor tools, and co-design methods with adult end users to ensure that the information delivered is appropriate to support adults. Due to the recognized importance of effective education in achieving standardized HBPM and improving BP control, creating appropriate educational resources for key BP management behaviors such as HBPM should be considered a priority.

## Appendix

Multimedia Appendix 1 The guideline recommendations used for resource appraisal.

Multimedia Appendix 2 Development of the search strategy. Consumer advisors (n=6) and Google Trends data were used to develop the search strategy to identify web-based home blood pressure measurement resources. Search terms suggested by consumer advisors that also had a high probability of use on Google (January 1, 2012, to October 7, 2022) were used.

Multimedia Appendix 3 Home blood pressure measurement (HBPM) resource eligibility and appraisal form. HBPM resources were appraised for eligibility and appraised for alignment to HBPM guidelines, grade reading level, and end user involvement in development according to the questions in the form house on REDCap (Research Electronic Data Capture).

Multimedia Appendix 4 Incorrectly stated guideline recommendations. Resource information marked as "incorrectly stated" during resource appraisal "step 1 alignment to guideline recommendations" was recorded in the REDCap (Research Electronic Data Capture) appraisal framework.

Multimedia Appendix 5 Resources included in the study.

Multimedia Appendix 6 Search strategy and results.

Multimedia Appendix 7 Sydney Health Literacy Lab Editor results of home blood pressure measurement resources. All text within resources, including written text and transcripts of audio and video material, was input to the Sydney Health Literacy Lab Editor. Grade reading level was calculated using the Simple Measure of Gobbledygook method.

## Abbreviations

AI: artificial intelligence
BP: blood pressure
HBPM: home blood pressure measurement
REDCap: Research Electronic Data Capture
SHeLL Editor: Health Literacy Lab Editor

## Data Availability

The data that support the findings of this study are available from the corresponding author (NC) upon reasonable request. NC had full access to all data in the study and takes responsibility for its integrity and the data analysis.
