# Supplementary material for: Appropriateness of Web-Based Resources for Home Blood Pressure Measurement and Their Alignment With Guideline Recommendations, Readability, and End User Involvement: Environmental Scan of Web-Based Resources
Source: JMIR Infodemiology. 2025 Apr 3;5:e55248. doi: 10.2196/55248 (PMC12006778; doi:10.2196/55248)
Supplement: Multimedia Appendix 1 [file infodemiology_v5i1e55248_app1.docx]

**The guideline recommendations used for resource appraisal.**

|  | **Guideline publication location** | | | | | |
| --- | --- | --- | --- | --- | --- | --- |
|  | **America** | **Australia** | **Canada** | **Europe** | **International** | **Japan** |
| ***Acquiring the BP device*** |  |  |  |  |  |  |
| Use a validated BP measurement device for HBPM. | ✓ | ✓ | ✓ | ✓ | ✓ |  |
| Finger cuff BP measurement devices should not be used for HBPM. | ✓ | ✓ | ✓ | ✓ |  |  |
| ***Scheduling HBPM*** |  |  |  |  |  |  |
| On a day that HBPM is being conducted, BP should be measured in the morning and the evening. | ✓ | ✓ | ✓ | ✓ | ✓ | ✓ |
| ***Preparing for HBPM*** |  |  |  |  |  |  |
| Do not measure BP if uncomfortable, stressed or in pain. |  | ✓ |  |  |  |  |
| Measure BP before medication. |  | ✓ | ✓ | ✓ |  | ✓ |
| Measure BP before eating or 30 minutes or 2 hours after eating. |  | ✓ | ✓ | ✓ |  | ✓ |
| Measure BP after emptying the bladder. | ✓ | ✓ |  |  | ✓ | ✓ |
| Measure BP before exercise, or 30 minutes after exercise. |  | ✓ | ✓ | ✓ | ✓ |  |
| Measure BP before caffeine, or 30 minutes or 1 hour after caffeine. |  | ✓ | ✓ | ✓ | ✓ | ✓ |
| Measure BP before smoking, or 30 minutes or 1 hour after smoking. |  | ✓ | ✓ | ✓ | ✓ | ✓ |
| Have 5 minutes, or at least 5 minutes, of seated rest before measuring BP. |  | ✓ | ✓ |  | ✓ |  |
| ***Selecting and fitting the cuff*** | |  |  |  |  |  |
| Use an appropriately sized arm cuff for HBPM. | ✓ | ✓ |  | ✓ |  |  |
| The arm cuff should fit the arm within the accepted range indicated on the cuff. | ✓ | ✓ |  | ✓ |  |  |
| Fit the upper arm BP cuff to a bare arm. | ✓ | ✓ | ✓ | ✓ |  |  |
| ***Measurement conditions*** |  |  |  |  |  |  |
| Measure BP in a room at a comfortable temperature. |  | ✓ |  | ✓ | ✓ | ✓ |
| Measure BP with the arm fitted with the cuff supported. |  | ✓ | ✓ |  |  |  |
| Measure BP in a seated position. | ✓ | ✓ | ✓ | ✓ | ✓ | ✓ |
| Measure BP with both feet flat on the floor. | ✓ | ✓ |  | ✓ | ✓ |  |
| Measure BP with legs uncrossed. | ✓ | ✓ |  | ✓ | ✓ | ✓ |
| Measure BP with back supported. | ✓ | ✓ |  | ✓ | ✓ |  |
| Take two readings one minute apart at each HBPM sitting. |  | ✓ |  | ✓ | ✓ |  |
| ***Recording and reporting BP*** | |  |  |  |  |  |
| Average the BP readings taken over a seven-day period, discarding the first day. |  | ✓ | ✓ | ✓ | ✓ |  |
| Take a copy of home BP readings to a doctor |  | ✓ |  |  |  |  |
