# Supplementary material for: Appropriateness of Web-Based Resources for Home Blood Pressure Measurement and Their Alignment With Guideline Recommendations, Readability, and End User Involvement: Environmental Scan of Web-Based Resources
Source: JMIR Infodemiology. 2025 Apr 3;5:e55248. doi: 10.2196/55248 (PMC12006778; doi:10.2196/55248)
Supplement: Multimedia Appendix 2 [file infodemiology_v5i1e55248_app2.docx]

**Development of search strategy.** Consumer advisors (n=6) and Google Trends data were used to develop the search strategy to identify online HBPM resources. Search terms suggested by consumer advisors that also had a high probability of use on Google 1/1/2012 to 7/10/2022 were used.

| **Step 1. Search terms developed with consumer advisors (n=6)** | | |
| --- | --- | --- |
| **Search term suggested by consumer advisor (n=15)** | **Consolidated consumer advisor search term (n=9)** | |
| Home blood pressure measure | Home blood pressure measurement. | |
| Home blood pressure measurement. |  |  |
| Blood pressure measurement at home |  |  |
| home measurement AND Blood pressure |  |  |
| Blood pressure AND home |  |  |
| How to measure blood pressure at home | How to measure blood pressure at home | |
| How to measure your own blood pressure at home |  |  |
| How to take your blood pressure | How to take your blood pressure | |
| How to measure blood pressure correctly at home | How to measure blood pressure correctly at home | |
| Accurate blood pressure measurement technique | Accurate blood pressure measurement technique | |
| Are blood pressure self-checks accurate? | Are blood pressure self-checks accurate? | |
| Optimum time to monitor blood pressure | NA | |
| Certified blood pressure monitors | Certified blood pressure monitors | |
| Accurate blood pressure monitor | Accurate blood pressure monitor | |
| Most reliable home blood pressure kits | Most reliable home blood pressure kits | |
| **Step 2. Search terms with the highest probability of use according to Google Trends.** | | |
| **Consumer advisor search term** | **Associated Google Trend search term (top 5)** | **Relative probability of use in Google (%)** |
| Home blood pressure measurement. | blood pressure monitor | 100 |
|  | home blood pressure monitoring | 52 |
|  | blood pressure chart | 30 |
|  | how to take blood pressure at home | 16 |
| How to measure blood pressure at home | how to measure high blood pressure | 100 |
|  | how to measure bp at home | 65 |
|  | how to check blood pressure | 63 |
|  | how to measure heart rate | 60 |
|  | blood pressure monitor | 56 |
|  | normal blood pressure | 54 |
|  | how to monitor blood pressure at home | 52 |
|  | low blood pressure | 52 |
|  | how to measure blood pressure without equipment | 41 |
|  | how to take blood pressure at home | 40 |
|  | blood pressure cuff | 38 |
|  | high blood pressure symptoms | 24 |
|  | how to measure blood sugar at home | 22 |
|  | how to measure blood pressure manually | 22 |
|  | blood pressure chart | 15 |
|  | symptoms of high blood pressure | 12 |
|  | low blood pressure symptoms | 12 |
|  | signs of high blood pressure | 11 |
|  | how to check blood pressure at home without equipment | 10 |
|  | how to measure blood pressure with sphygmomanometer | 9 |
|  | how to read blood pressure | 7 |
|  | how to check blood pressure by hand | 6 |
| How to take your blood pressure | high blood pressure | 100 |
|  | what is blood pressure | 80 |
|  | lower blood pressure | 70 |
|  | how to lower your blood pressure | 70 |
|  | how to lower blood pressure | 70 |
|  | when to take your blood pressure | 62 |
|  | when to take blood pressure | 62 |
|  | how do you take your blood pressure | 56 |
|  | how to take my blood pressure | 43 |
|  | how long does it take to lower your blood pressure | 37 |
|  | how long does it take to lower blood pressure | 37 |
|  | low blood pressure | 34 |
|  | normal blood pressure | 32 |
|  | blood pressure medication | 31 |
|  | what is high blood pressure | 30 |
|  | how to raise your blood pressure | 28 |
|  | how to raise blood pressure | 28 |
|  | how to take your heart rate | 25 |
|  | good blood pressure | 24 |
|  | how to take pulse | 24 |
|  | how to take your pulse | 24 |
|  | what to take for high blood pressure | 23 |
|  | how to take your own blood pressure | 22 |
|  | how to lower high blood pressure | 22 |
|  | how to take blood pressure at home | 21 |
| Accurate blood pressure monitor | are wrist blood pressure monitors accurate | 100 |
|  | accurate blood pressure monitor | 78 |
|  | blood pressure monitor | 78 |
|  | are home blood pressure monitors accurate | 47 |
|  | how accurate are wrist blood pressure monitors | 38 |
|  | wrist blood pressure monitor | 34 |
|  | omron blood pressure | 33 |
|  | omron | 33 |
|  | omron blood pressure monitors | 32 |
|  | best blood pressure monitors | 28 |
|  | how accurate are home blood pressure monitors | 23 |
|  | home blood pressure monitor | 22 |
|  | omron blood pressure monitor | 21 |
|  | most accurate blood pressure monitor | 21 |
|  | best blood pressure monitor | 20 |
|  | blood pressure cuff | 20 |
|  | are wrist bp monitors accurate | 16 |
|  | cvs | 7 |
|  | walgreens | 6 |
|  | how to lower blood pressure | 4 |
|  | upper arm blood pressure monitor | 4 |
|  | are wrist blood pressure monitors as accurate as arm monitors | 4 |
|  | boots | 4 |
|  | systolic blood pressure | 3 |
|  | boots blood pressure monitor | 3 |
| How to measure blood pressure correctly at home | Insufficient Google Trends data. |  |
| Accurate blood pressure measurement technique | Insufficient Google Trends data. |  |
| Certified blood pressure monitors | Insufficient Google Trends data. |  |
| Are blood pressure self-checks accurate? | Insufficient Google Trends data. |  |
| Most reliable home blood pressure kits | Insufficient Google Trends data. |  |
| **Step 3. Final search terms (n=7).** | | |
| Home blood pressure measurement | | |
| Home blood pressure monitoring | | |
| How to measure blood pressure at home | | |
| How to take your blood pressure | | |
| How to take blood pressure at home | | |
| How to check blood pressure at home | | |
| How to monitor blood pressure at home | | |

NA: not applicable
