# Supplementary material for: Appropriateness of Web-Based Resources for Home Blood Pressure Measurement and Their Alignment With Guideline Recommendations, Readability, and End User Involvement: Environmental Scan of Web-Based Resources
Source: JMIR Infodemiology. 2025 Apr 3;5:e55248. doi: 10.2196/55248 (PMC12006778; doi:10.2196/55248)
Supplement: Multimedia Appendix 3 [file infodemiology_v5i1e55248_app3.docx]

**HBPM resource eligibility and appraisal form.** HBPM resources were appraised for eligibility and appraised for alignment to HBPM guidelines, grade reading level and end-user involvement in development according to the questions in the form house on REDCap.

|  | Name of reviewer | 1, EC  2, SC |
| --- | --- | --- |
|  | **Section 1: Eligibility screening** | |
| 1.1 | Date of inclusion or exclusion from the scoping review. | [DD/MM/YYYY] |
| 1.2 | Search term/s used to extract this resource:  *Multiple choice answer* | 1, How to take your blood pressure  2, How to check blood pressure at home  3, How to take blood pressure at home  4, Home blood pressure monitoring  5, How to measure blood pressure at home  6, How to monitor blood pressure at home  7, Home blood pressure measurement |
| 1.3 | Resource URL | [free text] |
| 1.4 | Title of resource | [free text] |
| 1.5 | Website domain name | [free text] |
| 1.6 | Resource is freely accessible to general public with no paywall, subscription fee. | 1, Yes  2, No |
| 1.7 | Resource is in English | 1, Yes  2, No |
| 1.8 | Resource is presented in first page of search results | 1, Yes  2, No |
| 1.9 | Resource is relevant to HBPM (the resource uses language that refers to HBPM, or an individual self-measuring blood pressure) | 1, Yes  2, No |
|  | *Resource eligible for appraisal if marked as ‘yes’ for eligibility questions 1.6, 1.7, 1.8 and 1.9.* | |
|  | **Section 2: Resource appraisal**  ***Resource characterisation*** | |
| 2.1 | Date of reviewer performing appraisal | [date] |
| 2.2 | Is the resource available in additional languages other than English | 1, Yes  2, No |
| 2.2.1 | If yes, please select the language/s:  *Multiple choice answer* | 1, Mandarin  2, Spanish  3, Hindi  4, Japanese  5, Italian  6, French  7, Arabic  8, Portugese  9, Indonesion  10, Russian  11, German  12, Other |
| 2.2.2 | If other, please specify: | [free text] |
| 2.3 | Is the publication date of the resource specified? | 1, Yes  2, No |
| 2.3.1 | If yes, what is the publication date of the resource. | [free text] |
| 2.4 | Is the last review date of the resource specified? | 1, Yes  2, No |
| 2.4.1 | If yes, what is the last review date of the resource. | [free text] |
| 2.5 | What is the target audience of the resource? | 1, Community member  2, Health professional (e.g. medical researcher, doctor, nurse, academic)  3, Other  4, Not specified |
| 2.5.1 | If other, please specify: | [free text] |
| 2.6 | Is this resource a guideline? | 1, Yes  2, No |
| 2.7 | Is this resource an expert consensus statement? | 1, Yes  2, No |
| 2.8 | Is this resource a scientific statement? | 1, Yes  2, No |
| 2.9 | *If no to 2.6, 2.7 or 2.8:* What is the primary purpose of the resource | 1, Commercial  2, Informative  3, Other |
| 2.9.1 | If other, please specify: | [free text] |
| 2.10 | What is the format of the resource?  *Multiple choice answer* | 1, Webpage  2, Document/Report  3, Advertisement (e.g. Ebay page)  4, Blog/ forum  5, Scientific paper  6, Video  7, News article/media report  8, Other |
| 2.10.1 | If other, please specify | [free text] |
| 2.11 | Which mode/s of communication are used within the resource?  *Multiple choice answer* | 1, Written text  2, Audio  3, Visual (image/s)  4, Video |
| 2.11.1 | *If audio or video selected in 2.11:* Are subtitles available? | 1, Yes  2, No |
| 2.11.2 | *If written text is selected in 2.11:* Does it state that read aloud is not available? | 1, Yes  2, No |
| 2.11.3 | *If written text is selected in 2.11:* Are visual descriptions available for images included in the resource? | 1, Yes  2, No |
| 2.12 | Is a printable version of the resource available? | 1, Yes  2, No |
| 2.13 | Does the resource provide a link to share to other online platforms? e.g. Facebook, LinkedIn, Endnote, email | 1, Yes  2, No |
| 2.14 | Has the resource been reviewed by a medical professional? | 1, Yes  2, No |
| 2.15 | Who are the medical reviewers of the resource? | [free text] |
| 2.16 | What is/are the name/s of the publishing organisation/s of the resource? | [free text] |
| 2.17 | What is/are the type/s of publishing organisation/s?  *Multiple choice answer* | 1, Commercial entity  2, Non-Government organisation  3, Journal  4, Government body e.g. .gov.au  5, Funding body e.g. NHMRC, RHHRF  6, Not for profit oganisation e.g. Mayo-clinic, Heart Foundation  7, Scientific society/professional body e.g. HBPRCA, AHA, RACGP  8, University  9, Self-published e.g. blog  10, Website e.g. WebMD  11, Unknown  12, Other |
| 2.17.1 | If other, please specify | [free text] |
| 2.18 | Where was the resource published?  *Single choice answer* | 1, Australia  2, North America  3, South America  4, Europe  5, Asia  6, Africa  7, Unknown |
| 2.19 | Has the resource been endorsed by an external party? | 1, Yes  2, No  3, Does not specify |
| 2.19.1 | If yes, name the endorsing party/parties: | [free text] |
| 2.19.2 | If yes, select the type of endorsing party:  *Multiple choice answer* | 1, Commercial entity  2, Non-Government organisation e.g. Mayo-clinic, Heart Foundation  3, Government body  4, Funding body e.g. NHMRC, RHHRF  5, Scientific society/professional body e.g. HBPRCA, AHA, RACGP  6, University  7, Hospital  8, Research organisation (e.g. Menzies)  9, Unknown  10, Other |
| 2.19.3 | If other, please specify: | [free text] |
| 2.20 | Does the resource present evidence of co-design with consumers/community members/the public? | 1, Yes  2, No |
|  | ***Alignment to guideline recommendations for HBPM*** | |
| 2.21 | The resource states that a BP machine can be bought for HBPM. | 1, Yes, the resource states this correctly  2, No, the resource states this incorrectly  3, No, the resource does not mention this at all |
| 2.21.1 | If yes, from where does it specify to buy a BP device  *Multiple choice answer* | 1, Chemist  2, Pharmacy  3, Online  4, Medical equipment supplier  5, General electronics retailer  6, Other  7, It does not specify |
| 2.21.2 | If other, please specify | [free text] |
| 2.22 | The resource states that a BP machine can be hired for HBPM. | 1, Yes, the resource states this correctly  2, No, the resource states this incorrectly  3, No, the resource does not mention this at all |
| 2.23 | The resource states that a BP machine can be borrowed for HBPM. | 1, Yes, the resource states this correctly  2, No, the resource states this incorrectly  3, No, the resource does not mention this at all |
| 2.24 | If the resource states 2.21, 2.22 or 2.23 incorrectly, please specify what the resource states: | |
| 2.25 | The BP device used for HBPM must be validated | 1, Yes, the resource states this correctly  2, No, the resource states this incorrectly  3, No, the resource does not mention this at all |
| 2.25.1 | If the resource states the above incorrectly, please specify what the resource states: | [free text] |
| 2.26 | The resource states that finger cuff BP devices should not be used for HBPM. | 1, Yes, the resource states this correctly  2, No, the resource states this incorrectly  3, No, the resource does not mention this at all |
| 2.26.1 | If the resource states this incorrectly, please specify what the resource states: | [free text] |
| 2.27 | The resource states that an appropriately sized cuff must be used for HBPM. | 1, Yes, the resource states this correctly  2, No, the resource states this incorrectly  3, No, the resource does not mention this at all |
| 2.27.1 | If the resource states this incorrectly, please specify what the resource states: | [free text] |
| 2.28 | The resource states that the appropriate cuff size of the BP device is selected because of the following reason/s:  *Multiple choice answer* | 1, Fits the arm within the accepted range indicated on the cuff  2, Select the cuff size as recommended by its manufacturer/device instruction  3, Bladder width should be close to 40% of arm circumference and bladder length should cover 80 - 100% of arm circumference.  4, Determined according to the individual's arm circumference  5, Other  6, Does not say |
| 2.28.1 | If other, please specify: | [free text] |
| 2.29 | The resource states that in patients with a very large arm circumference a conic shaped upper-arm cuff should be used. | 1, Yes, the resource states this correctly  2, No, the resource states this incorrectly  3, No, the resource does not mention this at all |
| 2.29.1 | If the resource states this incorrectly, please specify what the resource states: | [free text] |
| 2.30 | The resource states that BP should be measured in the morning and the evening. | 1, Yes, the resource states this correctly  2, No, the resource states this incorrectly  3, No, the resource does not mention this at all |
| 2.30.1 | If the resource states this incorrectly, please specify what the resource states: | [free text] |
| 2.31 | The resource states that the upper arm BP cuff should be fitted to a bare arm | 1, Yes, the resource states this correctly  2, No, the resource states this incorrectly  3, No, the resource does not mention this at all |
| 2.31.1 | If the resource states this incorrectly, please specify what the resource states: | [free text] |
| 2.32 | The resource states that the arm fitted with the upper arm BP device should be:  *Multiple choice answer* | 1, Supported (relaxed position).  2, Supported on a flat surface such as a table  3, Resting on a table with mid-arm at heart level  4, Other  5, Does not specify |
| 2.32.1 | If other, please specify | [free text] |
| 2.33 | The resource states that HBPM should be conducted in a room:  *Multiple choice answer* | 1, With a comfortable temperature  2, At room temperature  3, Other  4, Does not specify |
| 2.33.1 | If other, please specify: | [free text] |
| 2.34 | The resource states that HBPM should be conducted after:  *Multiple choice answer* | 1, 5 minutes of seated rest  2, At least 5 minutes of seated rest  3, 3-5 minutes of seated rest  4, 1-2 minutes of seated rest  5, Other  6, Does not specify |
| 2.35 | If other, please specify | [free text] |
| 2.36 | Not measure BP if uncomfortable, stressed or in pain. | 1, Yes, the resource states this correctly  2, No, the resource states this incorrectly  3, No, the resource does not mention this at all |
| 2.36.1 | If the resource states this incorrectly, please specify what the resource states: | [free text] |
| 2.37 | Measure BP in a seated position | 1, Yes, the resource states this correctly  2, No, the resource states this incorrectly  3, No, the resource does not mention this at all |
| 2.37.1 | If the resource states this incorrectly, please specify what the resource states: | [free text] |
| 2.38 | Measure BP with both feet flat on the floor | 1, Yes, the resource states this correctly  2, No, the resource states this incorrectly  3, No, the resource does not mention this at all |
| 2.38.1 | If the resource states this incorrectly, please specify what the resource states: | [free text] |
| 2.39 | Measure BP with legs uncrossed | 1, Yes, the resource states this correctly  2, No, the resource states this incorrectly  3, No, the resource does not mention this at all |
| 2.39.1 | If the resource states this incorrectly, please specify what the resource states: | [free text] |
| 2.40 | Measure BP with back supported | 1, Yes, the resource states this correctly  2, No, the resource states this incorrectly  3, No, the resource does not mention this at all |
| 2.40.1 | If the resource states this incorrectly, please specify what the resource states: | [free text] |
| 2.41 | The resource states that HBPM should be conducted:  *Multiple choice answer* | 1, Before medication  2, Before anti-hypertensive medication  3, Before medication in both the morning and the evening  4, Other  5, Does not specify |
| 2.41.1 | If other, please specify: | [free text] |
| 2.41.2 | If the resource states this incorrectly, please specify what the resource states: | [free text] |
| 2.42 | The resource states that HBPM should be conducted:  *Multiple choice answer* | 1, Before eating  2, No sooner than 30 minutes after eating  3, Before eating breakfast  4, No sooner than 2 hours after eating dinner  5, Before eating dinner  6, Other  7, Does not specify |
| 2.42.1 | If other, please specify: | [free text] |
| 2.43 | The resource states that HBPM should be conducted:  *Multiple choice answer* | 1, After voiding  2, After emptying one's bladder  3, Other  4, Does not specify |
| 2.43.1 | If other, please specify: | [free text] |
| 2.44 | The resource states that HBPM should be conducted:  *Multiple choice answer* | 1, Before exercise  2, No sooner than 30 minutes after exercise  3, Other  4, Does not specify |
| 2.44.1 | If other, please specify: | [free text] |
| 2.45 | The resource states that HBPM should be conducted:  *Multiple choice answer* | 1, Before consuming coffee  2, No sooner than 30 minutes after consuming coffee  3, No sooner than 1 hour after consuming coffee  4, Other  5, Does not specify |
| 2.45.1 | If other, please specify: | [free text] |
| 2.46 | The resource states that HBPM should be conducted:  *Multiple choice answer* | 1, Before smoking  2, No sooner than 30 minutes after smoking  3, No sooner than 1 hour after smoking  4, Other  5, Does not specify |
| 2.46.1 | If other, please specify: | [free text] |
| 2.47 | The resource states that when taking BP measurements:  *Multiple choice answer* | 1, Two BP readings should be taken  2, Two BP readings should be taken, 1 min apart  3, Other  4, Does not specify |
| 2.47.1 | If other, please specify: | [free text] |
| 2.48 | The resource states that when calculating BP from measurements collected over the entire HBPM duration:  *Multiple choice answer* | 1, The first day of measurements should be discarded.  2, If the first day of measurements is discarded, then the period of measurement (i.e. 7 days) should be extended by one day (i.e. 8 days)  3, Other  4, Does not specify |
| 2.48.1 | If other, please specify: | [free text] |
| 2.49 | The resource states that a copy of BP readings should be taken to the doctor when HBPM is completed. | 1, Yes, the resource states this correctly \| 2, No, the resource states this incorrectly \| 3, No, the resource does not mention this at all |
| 2.49.1 | If the resource states this incorrectly, please specify what the resource states: | [free text] |
| ***Section 3: Grade reading level***  *Data output from Sydney Health Literacy Lab Editor* | | |
| 3.1 | What is the grade reading score of the resource text? | [free text] |
| 3.2 | How many words that are > 2 syllables were used within the resource text? | [number] |
| 3.3 | How many sentences that are too long were used within the resource text? | [number] |
| 3.4 | What is the text complexity score of the resource? | [free text] |
| 3.5 | How many words or phrases were used in the resource text which have alternative meanings in SHeLL's thesaurus? | [number] |
| 3.6 | Have the words which were identified as having >1 possible meaning been defined in the resource text?  *Single answer question* | 1, Yes, all of them  2, Yes, some of them  3, No, none of them  4, No words with >1 meaning were used |
| 3.7 | How many uncommon words were used within the resource? | [number] |
| 3.8 | How many acronyms were used within the resource? | [number] |
| 3.9 | Have the acronyms used within the text been defined in the first instance?  *Single answer question* | 1, Yes, all of them  2, Yes, some of them  3, No, none of them  4, No acronyms were used |
| 3.10 | How many times was passive voice used in the resource? | [number] |
| 3.11 | How many paragraphs are too long? | [number] |
