# Supplementary material for: Appropriateness of Web-Based Resources for Home Blood Pressure Measurement and Their Alignment With Guideline Recommendations, Readability, and End User Involvement: Environmental Scan of Web-Based Resources
Source: JMIR Infodemiology. 2025 Apr 3;5:e55248. doi: 10.2196/55248 (PMC12006778; doi:10.2196/55248)
Supplement: Multimedia Appendix 4 [file infodemiology_v5i1e55248_app4.docx]

**Incorrectly stated guideline recommendations**. Resource information that was marked as ‘incorrectly stated’ during resource appraisal ‘step 1 alignment to guideline recommendations’ was recorded in the REDCap appraisal framework.

| **Guideline recommendation** | **Resource information** | **Justification for misalignment** |
| --- | --- | --- |
| ***Acquiring the BP device*** |  |  |
| Use a validated BP measurement device for HBPM. | *"Remember that many devices have not been formally tested for accuracy, so use them as a guide only."* (Resource ID 5). | Does not recommend for or against the use of a validated device. |
|  | *Resource provides link to list of validated devices* (Resource ID 7). | Does not recommend for or against the use of a validated device. |
| Finger cuff BP measurement devices should not be used for HBPM. | *"Tests show that finger and wrist devices do not always provide correct measurements. These devices are sensitive to placement and body temperature. They also are expensive and can cost more than $100."* (Resource ID 21). | Does not recommend for or against the use of a finger cuff device. |
| ***Scheduling HBPM*** |  |  |
| On a day that HBPM is being conducted, BP should be measured in the morning and the evening. | *"It is recommended that you measure your own blood pressure with an automatic blood pressure monitor first thing in the morning, while you are still in bed"* (Resource ID 20). | Does not state that BP should be measured in the evening. |
| ***Preparing for HBPM*** |  |  |
| Do not measure BP if uncomfortable, stressed or in pain. | *“Try to be calm and not think about stressful things.”* (Resource ID 3). | Does not state to avoid measuring BP when stressed. |
|  | *"Make sure you are relaxed and comfortable. If you are anxious or uncomfortable, your blood pressure will rise temporarily."* (Resource ID 4) | Describes impact of stress on BP, but does not state to avoid measuring BP when stressed. |
|  | *"Find a comfortable place to sit”* (Resource ID 19) | Does not state to avoid measuring BP when uncomfortable. |
|  | *"Sit in a comfortable position, with your legs and ankles uncrossed and your back supported."* (Resource ID 21) |  |
|  | *"Make sure you're comfortable"* (Resource ID 22) |  |
| Measure BP before medication. | *"Avoid certain medication when taking blood pressure"* (Resource ID 8). | Does not state to avoid taking medication before measuring BP. |
| Measure BP before eating or 30 minutes or 2 hours after eating. | *"Record special circumstances like any recent exercise, meal or stressful event"* (Resource ID 8). | Does not state to avoid eating before BP measurement. |
| Measure BP after emptying the bladder. | *"You should also avoid measuring your blood pressure when you need to use the toilet."* (Resource ID 17). | Does not state to go to the toilet before measuring BP. |
| Measure BP before exercise, or 30 minutes after exercise. | *"If you smoked, drank alcohol or caffeine, or exercised within 30 minutes of having your blood pressure measured, your reading might be higher."* (Resource ID 2). | States the impact of exercise on BP. |
|  | *"There are a number of factors that can lead to incorrect readings, like recent physical activity, time of day, arm position or user error can all skew your results."* (Resource ID 13). |  |
|  | *"Avoid exercise when taking blood pressure"* (Resource ID 8). | Does not state to exercise smoking before BP measurement. |
| Measure BP before caffeine, or 30 minutes or 1 hour after caffeine. | *"If you smoked, drank alcohol or caffeine, or exercised within 30 minutes of having your blood pressure measured, your reading might be higher."* (Resource ID2) | States the impact of caffeine on BP. |
|  | *"Avoid caffeine when taking blood pressure"* (Resource ID 8) | Does not state to avoid caffeine before BP measurement. |
| Measure BP before smoking, or 30 minutes or 1 hour after smoking. | *"If you smoked, drank alcohol or caffeine, or exercised within 30 minutes of having your blood pressure measured, your reading might be higher."* (Resource ID 2). | States the impact of smoking on BP. |
|  | *"Avoid smoking when taking blood pressure"* (Resource ID 8). | Does not state to avoid smoking before BP measurement. |
| Have 5 minutes, or at least 5 minutes of seated rest before measuring BP. | *"Rest in a chair next to a table for 5 to 10 minutes"* (ID 8). | Alternate duration of seated rest. |
|  | *“3-5 minutes of seated rest”* (Resource ID 9, Resource ID 19). |  |
|  | *"Rest 3 to 5 minutes before taking your blood pressure and a few extra minutes if you've recently been very active."* (Resource ID 9). |  |
|  | *"Rest for 15 minutes"* (Resource ID 10). |  |
|  | *"Sit in a comfortable position, without distractions and refrain from talking or moving for a few minutes before taking the reading."* (Resource ID 16). |  |
|  | *"Rest for 3 to 5 minutes"* (Resource ID 21). | Does not state to sit for rest, alternate duration of rest. |
| ***Selecting and fitting the cuff*** |  |  |
| Use an appropriately sized arm cuff for HBPM. | *“Upper arm style monitors generally come with a medium sized cuff as standard. While this will be suitable for the majority of users, if you've got exceptionally large or small arms then look for one which has different sized cuffs available.”* (Resource ID 13). | States that alternate cuff sizes are available, but does not recommend to select certain cuff size according to arm size. |
| The arm cuff should fit the arm within the accepted range indicated on the cuff. | *“Select the cuff size as recommended by its manufacturer/device instruction”* (Resource ID 4, Resource ID 13, Resource ID 17, Resource ID 22). | Not specific instruction to use the accepted range on cuff. |
|  | *“Determined according to the individual's arm circumference”* (Resource ID 6, Resource ID 10, Resource ID 13, Resource ID 14, Resource ID 21, Resource ID 22). | Does not recommend using range indicated on cuff. |
|  | *"Ask your health care provider what cuff size you need."* (Resource ID 3). | Not the recommended method to determine cuff size. |
|  | *"The bladder inside the cuff should encircle 80% of the top of the arm"* (Resource ID 4). |  |
|  | *"The inflatable bladder of the cuff should cover 80-100 % of the individual's arm circumference."* (Resource ID 14) |  |
|  | *"You should be able to comfortably slip one finger between your arm and the cuff when it's deflated."* (Resource ID 9). |  |
|  | *"To find the right size cuff for you, measure your arm's circumference halfway between your shoulder and elbow while standing with your arm hanging at your side. A circumference of 18-22cm requires a small cuff, 22-32cm requires a medium cuff, and above 32cm you'll need a large cuff - but check the measurements against the manufacturer's instructions on the product you intend to buy."* (Resource ID 13) |  |
|  | *"A circumference of 18-22cm requires a small cuff, 22-32cm requires a medium cuff, and above 32cm you'll need a large cuff - but check the measurements against the manufacturer's instructions on the product you intend to buy."* (Resource ID 22). |  |
|  | *"In order to avoid a false reading that's too low (with a cuff that's too big) or too high (with a cuff that's too small), make sure the inflatable part of an arm cuff covers about 40% of the circumference of your upper arm and about 80% of the area from your elbow to shoulder"* (Resource ID 16) | Provides impact of incorrectly sized cuff on BP reading and states alternate method of selecting correct cuff size. |
| Fit the upper arm BP cuff to a bare arm. | *"Roll up the sleeve on your arm or remove any tight-sleeved clothing"* (Resource ID 8). | Unclear and doesn’t specifically state to have a bare arm. |
|  | *Video demonstration of cuff applied to a bare arm, but no instruction.* (Resource ID 10). |  |
|  | "Do not wear any tight or restrictive clothing around the arm you are measuring your blood pressure in. For example, you should avoid rolling up tight shirt sleeves." (Resource ID 17). |  |
| ***Measurement conditions*** |  |  |
| Measure BP in a room at a comfortable temperature. | *"Avoid cold temperatures when measuring blood pressure"* (Resource ID 8) | Does not state which temperature the room should be at. |
|  | *"No extreme temperatures"* (Resource ID 11, Resource ID 15) |  |
| Measure BP with the arm fitted with the cuff supported, or supported at heart level. | NA | NA |
| Measure BP in a seated position. | NA | NA |
| Measure BP with both feet flat on the floor. | NA | NA |
| Measure BP with legs uncrossed. | NA | NA |
| Measure BP with back supported. | *"Sit with your back straight"* (Resource ID 10). | Does not mention to rest back against a support. |
|  | *"Secure the cuff by threading the cuff end through the metal loop and slide the cuff onto your left arm, ensuring you sit straight"* (Resource ID 1). |  |
| Take two readings one minute apart at each HBPM sitting. | *"Take at least two readings, 1 or 2 minutes apart."* (Resource ID 2). | Alternate number of BP readings and duration of rest between BP readings. |
|  | *“Wait 1 to 3 minutes after the first reading, and then take another.”* (Resource ID 3). |  |
|  | *"Take three measurements, 1-2 minutes apart".* (Resource ID 4) |  |
|  | *"Record your blood pressure at least twice each time; take the readings 1 minute apart"* (Resource ID 5) | Alternate number of BP readings. |
|  | *"If you did not get an accurate reading, DO NOT inflate the cuff again right away. Wait one minute before repeating the measurement."* (Resource ID 8) | Reason for additional measurement is not stated in guideline recommendation. |
|  | *"Take at least two readings every time to make sure they're correct. The readings should be within a few numbers of each other."* (Resource ID 9) | Alternate number of BP readings and reason for additional measurement is not stated in guideline recommendation. |
|  | *“Two readings should be taken at least one minute apart”* (Resource ID 14). | Alternate duration of rest between BP readings. |
|  | *"Rest quietly and wait about one to two minutes before taking another measurement."* (Resource ID 19) |  |
|  | *"If you need to repeat the measurement, wait 2 to 3 minutes before starting."* (Resource ID 21) | Not specific about the number of BP readings to take and alternate duration of rest between BP readings. |
|  | *"Take two measurements within a couple of minutes of each other. If they differ by more than 10mmHg a difference of 15mmHg), take a third measurement."* (Resource ID 22). | Reason for additional measurement is not stated in guideline recommendation. |
|  | *"On each occasion take a minimum of two readings, leaving at least a minute between each. If the first two readings are very different, take 2 or 3 further readings."* (Resource ID 23). | Reason for additional measurement is not stated in recommendation, non-specific about the number of additional BP readings. |
| ***Recording and reporting BP*** |  |  |
| Average the BP readings taken over a seven-day period, discarding the first day. | *"The average measurement over the monitoring period is used to determine the patient's underlying blood pressure."* (Resource ID 12). | Does not state to discard the first day of BP measurement. |
| Take a copy of home BP readings to a doctor. | *"If you are concerned about your blood pressure numbers, talk to your health care team. They can help you make a plan to manage high blood pressure."* (Resource ID 2). | Does not specify which healthcare practitioner to report home BP readings to. |
