# Supplementary material for: Appropriateness of Web-Based Resources for Home Blood Pressure Measurement and Their Alignment With Guideline Recommendations, Readability, and End User Involvement: Environmental Scan of Web-Based Resources
Source: JMIR Infodemiology. 2025 Apr 3;5:e55248. doi: 10.2196/55248 (PMC12006778; doi:10.2196/55248)
Supplement: Multimedia Appendix 5 [file infodemiology_v5i1e55248_app5.docx]

**Resources included in study.**

| **Resource number** | **Title** | **Publishing organisation** | **Location of publication** | **URL** |
| --- | --- | --- | --- | --- |
| 1 | Check your blood pressure | Heart Research Institute | Australia | https://www.hri.org.au/check-your-blood-pressure |
| 2 | Measure Your Blood Pressure | Centers for Disease Control and Prevention | United States of America | https://www.cdc.gov/bloodpressure/measure.htm |
| 3 | Get the most out of home blood pressure monitoring | Mayo Clinic | United States of America | https://www.mayoclinic.org/diseases-conditions/high-blood-pressure/in-depth/high-blood-pressure/art-20047889 |
| 4 | How to measure your blood pressure at home | Blood Pressure UK | Europe | https://www.bloodpressureuk.org/your-blood-pressure/how-to-lower-your-blood-pressure/monitoring-your-blood-pressure-at-home/how-to-measure-your-blood-pressure-at-home/ |
| 5 | Measuring your blood pressure at home | Heart Foundation | Australia | https://resources.heartfoundation.org.au/images/uploads/publications/Measuring-your-blood-pressure-at-home.PDF |
| 6 | Monitoring Your Blood Pressure at Home | American Heart Association | United States of America | https://www.heart.org/en/health-topics/high-blood-pressure/understanding-blood-pressure-readings/monitoring-your-blood-pressure-at-home |
| 7 | How to accurately measure blood pressure at home | American Heart Association | United States of America | https://www.heart.org/en/news/2020/05/22/how-to-accurately-measure-blood-pressure-at-home |
| 8 | Checking Your Blood Pressure at Home | WebMD | United States of America | https://www.webmd.com/hypertension-high-blood-pressure/guide/hypertension-home-monitoring |
| 9 | How to Check Your Blood Pressure at Home | Healthline | United Stated of America | https://www.healthline.com/health/how-to-check-blood-pressure-by-hand |
| 10 | How To Take Blood Pressure Correctly | OMRON | United States of America | https://www.youtube.com/watch?v=iEwqy3lzK0c |
| 11 | How to measure home blood pressure: Recommendations for healthcare professionals and patients | Australian Family Physician | Australia | https://www.racgp.org.au/afp/2016/january-february/how-to-measure-home-blood-pressure-recommendations |
| 12 | Home monitoring of blood pressure | Australian Prescriber | Australia | https://www.nps.org.au/australian-prescriber/articles/home-monitoring-of-blood-pressure |
| 13 | How to buy the best blood pressure monitor | CHOICE | United States of America | https://www.choice.com.au/health-and-body/conditions/cardiovascular-disease/buying-guides/blood-pressure-monitors |
| 14 | Home Blood Pressure Monitoring | European Cardiology Review | Europe | https://www.ncbi.nlm.nih.gov/pmc/articles/PMC6159400/ |
| 15 | Home blood pressure monitoring: Australian Expert Consensus Statement | Journal of Hypertension | Europe | https://www.ncbi.nlm.nih.gov/pmc/articles/PMC4671913/ |
| 16 | Blood Pressure Monitors | Amcal+ | Australia | https://www.amcal.com.au/blood-pressure-monitors?#facet:&productBeginIndex:0&facetLimit:&orderBy:&pageView:grid&minPrice:&maxPrice:&sourcePage:&pageSize: |
| 17 | Home Blood Pressure Monitoring Explained | British Hypertension Society | Europe | https://bihsoc.org/wp-content/uploads/2017/09/Home_blood_pressure_monitoring_explained.pdf |
| 18 | Blood Pressure and your heart | Heart Foundation | Australia | https://www.heartfoundation.org.au/bundles/your-heart/blood-pressure-and-your-heart |
| 19 | Video: How to measure blood pressure using an automatic monitor | MayoClinic | United States of America | https://www.mayoclinic.org/diseases-conditions/high-blood-pressure/multimedia/how-to-measure-blood-pressure/vid-20084749 |
| 20 | High blood pressure | NeuRA | Australia | https://www.neura.edu.au/health/high-blood-pressure/?gclid=EAIaIQobChMI2KTNqIvm-gIV1H0rCh0_MAg_EAAYAyAAEgKJgfD_BwE |
| 21 | Blood Pressure Monitoring at Home | Family Doctor | United States of America | https://familydoctor.org/blood-pressure-monitoring-at-home/ |
| 22 | How to measure your blood pressure | CHOICE | Australia | https://www.choice.com.au/health-and-body/conditions/cardiovascular-disease/articles/steps-to-measure-blood-pressure?gclid=EAIaIQobChMI1dzK7OTq-gIVb5JmAh3--gUSEAAYBCAAEgIEF_D_BwE |
| 23 | Home Blood Pressure Diary | British Hypertension Society | Europe | https://bihsoc.org/wp-content/uploads/2017/09/Home_blood_pressure_diary.pdf |
| 24 | Automatic Blood Pressure Monitors | HMG Direct | Australia | https://www.hmgdirect.com.au/collections/automatic-blood-pressure-monitors?gclid=EAIaIQobChMIzZ3frsHm-gIVkp1LBR1vnwJDEAAYAiAAEgKawfD_BwE |
