# Supplementary material for: Appropriateness of Web-Based Resources for Home Blood Pressure Measurement and Their Alignment With Guideline Recommendations, Readability, and End User Involvement: Environmental Scan of Web-Based Resources
Source: JMIR Infodemiology. 2025 Apr 3;5:e55248. doi: 10.2196/55248 (PMC12006778; doi:10.2196/55248)
Supplement: Multimedia Appendix 6 [file infodemiology_v5i1e55248_app6.docx]

Duplicate resources removed
*n* = 59

Resources extracted across all seven search terms
*n* = 90

Resources assessed for inclusion
*n* = 31

Excluded resources
*n* = 7

- Not relevant to home blood pressure measurement = 7

Resources included for appraisal
*n* = 24

Resource identification

Eligibility screening

Included

Search terms:

- How to take your blood pressure = 13
- How to check blood pressure at home = 13
- How to take blood pressure at home = 14
- Home blood pressure monitoring = 11
- How to measure blood pressure at home = 13
- How to monitor blood pressure at home = 14
- Home blood pressure measurement = 12

**Search strategy and results.**
