# Supplementary material for: Appropriateness of Web-Based Resources for Home Blood Pressure Measurement and Their Alignment With Guideline Recommendations, Readability, and End User Involvement: Environmental Scan of Web-Based Resources
Source: JMIR Infodemiology. 2025 Apr 3;5:e55248. doi: 10.2196/55248 (PMC12006778; doi:10.2196/55248)
Supplement: Multimedia Appendix 7 [file infodemiology_v5i1e55248_app7.docx]

**Sydney Health Literacy Lab Editor results of HBPM resources.** All text within resources, including written text and transcripts of audio and video material, was input to the Sydney Health Literacy Lab Editor. Grade reading level was calculated using the Simple Measure of Gobbledygook method.

| Resource number | Readability | | | | Complex language | | | | Passive voice | Structure |
| --- | --- | --- | --- | --- | --- | --- | --- | --- | --- | --- |
|  | **Grade reading level** | **Words >2 syllables** | **Sentence >3 commas** | **Sentence >20 words** | **Complex language % score** | **Words or phrases with alternative** | **Uncommon words** | **Acronyms** |  | **Paragraph with >8 sentences or >150 words** |
| 1 | 10 | 13 | 0 | 1 | 7.8 | 2 | 5 | 0 | 0 | 0 |
| 2 | 8.8 | 61 | 0 | 16 | 7.6 | 19 | 34 | 11 | 2 | 0 |
| 3 | 10.9 | 187 | 0 | 17 | 18.1 | 118 | 86 | 3 | 4 | 0 |
| 4 | 9 | 116 | 0 | 22 | 8.2 | 58 | 43 | 9 | 5 | 0 |
| 5 | 11 | 130 | 0 | 12 | 12.6 | 45 | 52 | 14 | 8 | 0 |
| 6 | 11.9 | 139 | 1 | 11 | 18.5 | 76 | 61 | 9 | 10 | 0 |
| 7 | 11.8 | 102 | 2 | 12 | 13.9 | 41 | 44 | 4 | 3 | 0 |
| 8 | 9.4 | 95 | 0 | 8 | 18.9 | 38 | 120 | 0 | 5 | 0 |
| 9 | 10.5 | 212 | 1 | 23 | 15.5 | 70 | 154 | 10 | 15 | 0 |
| 10 | 9.9 | 14 | 0 | 1 | 12.1 | 8 | 8 | 0 | 0 | 0 |
| 11 | 14.4 | 274 | 1 | 25 | 26.3 | 123 | 136 | 72 | 39 | 0 |
| 12 | 15.1 | 341 | 1 | 22 | 29.3 | 204 | 170 | 0 | 27 | 1 |
| 13 | 13.5 | 331 | 1 | 44 | 18.5 | 162 | 168 | 11 | 13 | 0 |
| 14 | 17 | 849 | 2 | 75 | 32.5 | 391 | 421 | 196 | 56 | 8 |
| 15 | 16.4 | 740 | 8 | 75 | 29.3 | 381 | 370 | 189 | 76 | 1 |
| 16 | 12.9 | 201 | 1 | 24 | 19.3 | 105 | 89 | 0 | 4 | 0 |
| 17 | 11.3 | 192 | 0 | 27 | 11.9 | 101 | 53 | 5 | 12 | 0 |
| 18 | 9.8 | 127 | 0 | 19 | 13.3 | 66 | 69 | 2 | 7 | 0 |
| 19 | 10.2 | 29 | 0 | 2 | 15.7 | 11 | 25 | 0 | 1 | 0 |
| 20 | 14.9 | 55 | 1 | 9 | 24.8 | 36 | 34 | 3 | 8 | 0 |
| 21 | 8.9 | 158 | 0 | 2 | 17.5 | 72 | 166 | 0 | 7 | 0 |
| 22 | 11.5 | 191 | 6 | 27 | 13.4 | 81 | 97 | 9 | 9 | 0 |
| 23 | 11.5 | 32 | 0 | 4 | 10.5 | 12 | 8 | 3 | 1 | 0 |
| 24 | 14.9 | 17 | 0 | 1 | 36.7 | 12 | 10 | 1 | 0 | 0 |
| Mean | 11.9 | 192 | 1 | 20 | 18.0 | 93 | 101 | 23 | 13 | 0.4 |
| Target | 8 | Minimal | Minimal | Minimal | <20% | Minimal | Minimal | Minimal | <2 | Minimal |
